# Clinical Performance of a Multivariate Index Assay in Detecting Early-Stage Ovarian Cancer in Filipino Women

**DOI:** 10.3390/ijerph19169896

**Published:** 2022-08-11

**Authors:** Clarissa L. Velayo, Kareen N. Reforma, Renee Vina G. Sicam, Michele H. Diwa, Alvin Duke R. Sy, Ourlad Alzeus G. Tantengco

**Affiliations:** 1 Department of Physiology, College of Medicine, University of the Philippines Manila, Manila 1000, Philippines; 2 Department of Obstetrics and Gynecology, University of the Philippines—Philippine General Hospital, Taft Avenue, Manila 1000, Philippines; 3 Department of Pathology, College of Medicine, University of the Philippines Manila, Manila 1000, Philippines; 4 Department of Epidemiology and Biostatistics, College of Public Health, University of the Philippines, Manila 1000, Philippines; 5 College of Medicine, University of the Philippines Manila, Manila 1000, Philippines

**Keywords:** CA-125, IOTA-LR2, multivariate index assays, ovarian mass, Philippines, ultrasound

## Abstract

This study evaluated the clinical performance and overall utility of a multivariate index assay in detecting early-stage ovarian cancer in a Filipino population. This is a prospective cohort study among Filipino women undergoing assessment for an ovarian mass in a tertiary center. Patients diagnosed with early-stage ovarian cancer and who underwent a physical examination before level III specialist ultrasonographic and Doppler evaluation, multivariate index assay (MIA2G), and surgery for an adnexal mass were included in this study. Ovarian tumors were classified as high-risk for malignancy based on the IOTA-LR2 score. The ovarian imaging and biomarker results were correlated with the reference standard: surgico-pathologic findings. The MIA2G exhibited the best overall performance among individual classifiers with a sensitivity of 91.7% and NPV of 84.7%, with a concomitant higher sensitivity in early-stage disease, whether as an individual classifier (93.5%) or in serial combination with ultrasound (85.5%). The performance of biomarkers (specificity, positive predictive values, and AUROC) such as MIA2G and CA-125 significantly improved when combined with an ultrasound risk scoring approach (*p* < 0.01). MIA2G showed a higher sensitivity for detecting lesions among EOC and late-stage ovarian cancers than otherwise. The application of biomarkers for evaluating ovarian masses in our local setting is secondary to ultrasound but adopting multivariate index assays rather than CA-125 would increase the detection of early-stage ovarian cancers regardless of menopausal status. This is most relevant in areas where level III sonographers or gynecologic oncologists are limited and preoperative referrals to these specialists can improve the survival of our patients.

## 1. Introduction

Ovarian cancer is the 10th most common malignancy with the 5th highest mortality rate in the Philippines [1]. The general population’s risk factors for ovarian cancer are unclear, and screening has not been proven to affect mortality secondary to the disease. The age-standardized incidence and mortality rate per 100,000 females is 7.1 and 4.1 in High/Very High Human Development Index (HDI) countries, compared with 5.8 and 4.2 in Low/Medium HDI countries. Based on Globocan 2020 data, the Philippines has higher numbers. Incidence and mortality rates of 10.4 and 6.6, respectively, and a 5-year prevalence of 25.05 per 100,000 reveal ovarian cancer as a tremendous health burden in the country [2,3].

Early-stage ovarian cancer, defined here as stage I and II disease, entails removing the ovaries and uterus combined with peritoneal washings and biopsies, lymphadenectomy, and omentectomy for staging. In contrast, unilateral salpingo-oophorectomy may be offered to children and younger women. Staging the disease informs the need for treatment, mainly systemic chemotherapy and prognosis. On the other hand, the advanced-stage disease requires maximal cytoreductive surgery. These complex surgical procedures commonly require a multi-subspecialty approach to achieve zero or subcentimeter residual disease, directly affecting the patient’s prognosis. Hence, at the onset of evaluation, patients should have access to appropriate gynecologic oncological care [4,5].

Philippine General Hospital is the national university hospital. From its annual statistics of 2015–2020, 996 new ovarian cancer patients were seen at the outpatient clinic. Over this period, an average of 68.2% were above 40 years old. This is consistent with Philippine Data showing a steep increase in incidence in the 5th decade [1]. Thirty-eight percent of these patients were beyond surgico-pathologic stage I. However, 21% of patients were inadequately staged, mainly due to failure of referral to a gynecologic oncologist. The Philippine General Hospital is one of only two centers providing Gynecologic Oncology training in the country. Though primary surgery is performed in the Philippine General Hospital, it also receives post-operative referrals from other health facilities which may not have a gynecologic oncologist. There are currently 123 certified gynecologic oncologists all over the archipelago, the majority of whom practice in the National Capital Region. Suspicion of malignancy in an ovarian mass should warrant timely referral to these subspecialists.

The American College of Obstetrics and Gynecology recommends that women with an adnexal mass and any of these criteria: elevated CA-125, ultrasound findings of malignancy, ascites, a nodular or fixed pelvic mass, or evidence of abdominal or distant metastasis should be referred to a gynecologic oncologist. Moreover, the ACOG distinguishes between an elevated CA-125 in postmenopausal women from a very high CA-125 in perimenopausal women due to the poor specificity of this biomarker in the latter population [6].

There is no recognized standard recommendation for evaluating patients with an ovarian mass in the Philippines. Local guidelines suggest several evaluation methods, including the Risk of Malignancy Index (RMI), which considers menopausal status, ultrasound findings, and CA-125. Another is the Risk of Ovarian Malignancy Algorithm (ROMA), a tumor marker-based risk stratification system that uses CA-125, HE4, and menopausal status. Lastly, ultrasound models, such as the International Ovarian Cancer Tumour Analysis Assessment of Different NEoplasias in the adneXa (IOTA ADNEX), are used to triage patients [7]. Recently, multivariate index assays (MIA), such as MIA or Ova1^®^, and a second-generation multivariate index assay (MIA2G, Overa^®^) were introduced in the Philippines. These assays have been shown to have a high sensitivity for early-stage ovarian malignancy [8,9,10,11,12]. However, these tests are not yet maximized due to economic constraints and the lack of local experience [8,9,11,13].

The survival of an ovarian cancer patient with both early and advanced stages increases when a gynecologic oncologist performs the surgery compared with the general obstetrician-gynecologist [14]. This is attributed to the knowledge and application of surgical guidelines and the ability to eradicate the tumor [14,15]. Therefore, there is an urgent need for general obstetricians-gynecologists to be able to diagnose ovarian malignancy pre-operatively and refer to specialists trained in gynecologic malignancy care. Hence, this study determined the diagnostic performance of traditional tests (IOTA-LR2 and CA-125) and a multivariate index assay (MIA2G) in evaluating patients with early-stage ovarian cancer.

## 2. Materials and Methods

This is a prospective cohort study involving patients diagnosed with an ovarian mass in the University of the Philippines-Manila and Philippine General Hospital. The research protocol was approved by the University of the Philippines Manila Research Ethics Board (UPMREB) under UPMREB Code 2017-170-01 and was registered in the Philippine Health Research Registry (PHRR) under Registration ID PHRR180614-001843. Patients who met the following inclusion criteria were asked to participate: non-pregnant female, over 18 years of age at recruitment, considered for surgery, has not been previously evaluated by a gynecologic oncologist, and has not been diagnosed with cancer in the past five years. Upon voluntary written consent, each patient underwent a physical examination and interview prior to level III specialist ultrasonographic and Doppler evaluation.

### 2.1. Ovarian Ultrasound Test

The International Ovarian Tumour Analysis (IOTA)—Logistic Regression 2 (LR2) model was used to stratify patients into either a High Risk (HR) or a Low Risk (LR) group. The sonographic parameters used were the presence of ascites, the presence of papillations with detectable blood flow, irregular cyst walls, the presence of acoustic shadows, age, and a maximum diameter of the largest solid component. Patients were classified as high risk for this study if their IOTA-LR2 score was ≥10%. An obstetrician-gynecologist trained in ultrasound (KNR) conducted and reviewed all ultrasound tests.

### 2.2. MIA2G Test

For High Risk (HR) patients, those with an IOTA—LR2 estimated probability of malignancy greater than or equal to 10%, a second-generation multivariate index assay (MIA2G) was performed. The MIA2G (OVERA^®^) combined the levels of five protein biomarkers: Apolipoprotein A1 (APOA1), Human epididymis protein 4 (HE4), Cancer antigen-125 (CA-125), Follicle-stimulating hormone (FSH), and Transferrin (TRF) along with the woman’s menopausal status to generate a numerical risk score between 0.0 to 10.0 using a proprietary algorithm, OvaCalc^®^ software (Aspira Women’s Health, Inc., Austin, TX, USA). A cut-off score of 5.0 conferred a high risk of malignancy.

### 2.3. Statistical Analysis

After the data were extracted by the investigator from the patient charts, all the information was manually entered into an electronic spreadsheet file, and subsequent data processing and analysis were then carried out using the software Stata 13 (StataCorp LLC, College Station, TX, USA). Descriptive statistics such as the mean, median, standard deviation, and range were used to describe actual age in years. In contrast, frequency and percentage were used for the categorical variables such as pathological diagnosis, stage, sonographic findings, and risk stratification methods to provide an overview of the study population. Furthermore, the biomarker and imaging results were stratified based on menopausal status, pathologic diagnosis, and stage of malignancy.

The sensitivity, specificity, positive and negative predictive values, and correct classification rate in diagnosing early-stage ovarian cancer were computed for IOTA-LR2, CA-125, and MIA2G. The histopathologic findings served as the “gold” standard or variable representing the “true” presence of disease. The high prevalence of malignant subjects due to the study institution being a tertiary referral center necessitated an adjustment of the prevalence to a lower one agreed at 10% when computing the predictive values.

In addition, these diagnostic criteria were measured when serial testing was performed with the imaging-based procedures performed first, followed by select biomarkers such as the CA-125 and MIA2G.

## 3. Results

A total of 379 subjects were enrolled in the study and only 286 women were evaluable with ultrasonographic, MIA2G, and surgico-pathologic results. Of these, 153 (53.50%) were classified as benign and 133 (46.50%) were malignant. Among the patients with a malignant adnexal mass, 121 (90.98%) were epithelial ovarian cancers while 12 (9.02%) were non-epithelial ovarian cancers. Most patients with malignancy were diagnosed as early-stage ovarian cancer, i.e., stage I (60, 45.11%) and stage II ovarian cancer (6, 4.51%. Moreover, the majority (62.12%) of patients with early-stage ovarian cancer were pre-menopausal women (Table 1).

Histopathologic findings in early ovarian cancer stages I and II (*n* = 66) showed a predominance of epithelial ovarian cancers (87.88%) regardless of menopausal status (Table 2). Mucinous ovarian cancer was the most common subtype of EOC seen in this cohort of patients (34, 58.62%).

A total of 286 subjects were included in the study. Histopathologic findings in early ovarian cancer stages I and II (*n* = 66) showed a predominance of epithelial ovarian cancers (87.88%) regardless of menopausal status. Based on Table 3, MIA2G has higher sensitivity (91%, 95% CI: 84.8–95.3%) than CA-125 (76.7%, 95% CI: 68.6–83.6%) in identifying early-stage ovarian cancer. The performance of biomarkers (specificity, positive predictive values, and AUROC) such as MIA2G and CA-125 significantly improved when combined with an ultrasound risk scoring approach (*p* < 0.01). However, there was no sufficient evidence to suggest that the serial testing with MIA2G and IOTA-LR2 was statistically better than the seral testing with CA-125 and IOTA-LR2 in diagnosing early-stage ovarian cancer (*p* = 0.12).

When further stratification with menopausal status was evaluated (Table 4), the sensitivity of the different diagnostic tests was higher while the specificity was lower when used among postmenopausal than pre-menopausal women. However, there was no notable difference in the diagnostic performance (AUROC) of the different risk classification methods for early-stage ovarian cancer between pre-menopausal and postmenopausal participants in the study (*p* > 0.05).

Based on the findings in Table 5, it can be noted that there was a higher sensitivity for detecting lesions among epithelial and late-stage ovarian cancers than otherwise. However, there was no sufficient evidence to suggest statistical significance in such observations (*p* > 0.05).

## 4. Discussion

This study showed a 23.08% prevalence of early-stage ovarian cancer among Filipino patients with ovarian masses. The result of this study was comparable with previous reports that showed a 10%–30.5% prevalence of ovarian cancer among patients with ovarian masses [16,17,18,19]. Almost half of the ovarian cancer patients in this study had an early-stage disease at the time of diagnosis. Consistent with previous studies [20,21,22], we found that most patients with early-stage ovarian cancer were classified as EOC (87.88%). However, the most common histology type in our cohort was the mucinous subtype (58.62%). This contrasts with previous studies, which showed that serous carcinoma was the most common subtype of ovarian cancer with the highest occurrence and mortality in women [23,24,25,26]. Mucinous ovarian cancer is a rare tumor, accounting for 3% of all epithelial ovarian cancers [27]. It has a distinct mutational profile with a predominance for KRAS mutations rather than p53 and BRCA, the latter of which are more common in serous ovarian cancer [28].

Menopausal status is a known risk factor for ovarian cancer [29]. The majority of ovarian cancer is most commonly diagnosed after menopause between the ages of 60 to 64 years [29,30]. Our results showed that late-stage ovarian cancer was more common in postmenopausal than pre-menopausal women. However, pre-menopausal still comprised 39.04% of the patients with ovarian cancer in this study. Among early-stage ovarian cancer patients, 62.12% were pre-menopausal women. These results show the importance of highly sensitive and specific diagnostic tests for early-stage ovarian cancer in the Philippines. Accurate diagnosis of these patients can result in early referral to a gynecologic oncologist for a better prognosis and outcome for younger pre-menopausal women. Currently, referrals of patients with ovarian mass to gynecologic oncologists before surgery is still underutilized even in the presence of key determinants of increased risk for ovarian malignancy [31]. MIA2G may help clinicians in identifying patients at increased risk of having ovarian malignancy.

Ovarian cancer is a silent killer, with most patients (>85%) being diagnosed at advanced stages (Stage III/IV) [32]. The 5-year survival rate at these late stages is less than 25%, while the rates range from 50% to 95% at earlier stages of the disease [33]. Thus, early detection and management of ovarian cancer would significantly impact the prognosis of each patient. In the Philippines, CA-125 is still the most common biomarker combined with ultrasound imaging in the preoperative evaluation of ovarian masses, even though global reports show only a sensitivity of 57.1% and a positive predictive value of 24%, with half of stage I cases exhibiting normal preoperative CA-125 levels [6,34,35,36,37,38]. Our study findings also showed similarly low levels of accuracy (sensitivity of 76.7%, PPV of 66.7%).

The local introduction of a MIA2G would combine five biomarker results into a single “test value” related to the probability of malignancy and, therefore, have a single high-risk cut-off for all women, unlike CA-125 or other tests such as ROMA, or even the modification of ACOG (mod-ACOG) guidelines for the evaluation of suspicious pelvic masses [32]. The use of MIA2G in our population exhibited the best sensitivity individually (91.0%) and in series with ultrasound imaging (77.4%) for detecting early-stage cancers. When menopausal status was considered, the MIA2G exhibited the best sensitivity individually (86.3%) and in series with ultrasound imaging (69.9%) for early-stage pre-menopausal patients. It also exhibited the best sensitivity individually (96.7%) and in series with ultrasound imaging (86.7%) for early-stage postmenopausal patients. These findings agree with earlier validation studies by Coleman et al., where MIA2G testing had higher sensitivities for early-stage and each primary ovarian cancer subtype. Its specificity (69%) and PPV (40%) both proving superior to the MIA with 54% and 31%, respectively [11].

This is the first and the most extensive study in the Philippines to show the diagnostic accuracy of multivariate index assay alone and in combination with ovarian imaging for diagnosing early-stage ovarian cancer. This study also included a large sample size and had all types of ovarian cancer. However, the results of this study only apply to the study population and may not apply to other populations. Moreover, this study was not designed to address the cost-effectiveness of the MIA2G or its impact on gynecologic oncology referrals of the patients.

## 5. Conclusions

This study established MIA2G alone as a sensitive test for early-stage ovarian malignancy. Serial test with IOTA-LR2 and MIA2G improved the specificity of the test compared with MIA2G alone. The application of biomarker assays for evaluating ovarian masses in our local setting is secondary to ultrasound, but adopting multivariate index assays rather than CA-125 for adjunct testing would increase the detection of early-stage ovarian cancers regardless of menopausal status. This is most relevant in areas in the Philippines where level III expert sonographers or gynecologic oncologists are limited and preoperative referrals to these specialists can make the difference in prolonging the survival of our patients.

## Figures and Tables

**Table 1:** Demographic characteristics and pathologic results for evaluable subjects.

Characteristics	All Evaluable Women		
	Overall	Pre-Menopausal	Menopausal
**Number**	286	187	99
**Age in years**			
**Mean ± SD**	44.06 ± 13.14	36.62 ± 9.43	57.88 ± 5.83
**Median**	45	37	58
**Range**	18 to 78	18 to 52	42 to 78
**Number of pregnancies**			
**None**	77 (26.92%)	61 (32.62%)	16 (16.16%)
**1**	39 (13.64%)	34 (18.18%)	5 (5.05%)
**2**	38 (13.29%)	28 (14.97%)	10 (10.10%)
**3**	27 (9.44%)	16 (8.56%)	11 (11.11%)
**≥4**	89 (31.12%)	37 (19.79%)	52 (52.53%)
**Not specified**	16 (5.59%)	11 (5.88%)	5 (5.05%)
**Pathologic Diagnosis, *n* (%)**			
**Benign conditions**	153 (53.50%)	114 (60.96%)	39 (39.39%)
**Malignant conditions**	133 (46.50%)	73 (39.04%)	60 (60.61%)
**Epithelial ovarian cancer**	121 (90.98%)	64 (87.67%)	57 (95%)
**Non-epithelial ovarian**	12 (9.02%)	9 (12.33%)	3 (5%)
**Stage, *n* (%)**			
**Borderline**	31 (23.31%)	17 (23.29%)	14 (23.33%)
**I**	60 (45.11%)	39 (53.42%)	21 (35%)
**II**	6 (4.51%)	2 (2.74%)	4 (6.67%)
**III**	23 (17.29%)	8 (10.96%)	15 (25%)
**IV**	10 (7.52%)	4 (5.48%)	6 (10%)
**Not specified**	3 (2.26%)	3 (4.11%)	-

**Table 2:** Surgical pathology findings among stage I and II ovarian cancer patients.

Findings	All Evaluable	Pre-Menopausal	Post-Menopausal
**Number**	66	41	25
**Pathologic Diagnosis**			
**Epithelial ovarian cancer**	58 (87.88%)	36 (87.80%)	22 (88%)
**Serous**	12 (20.69%)	6 (16.67%)	6 (27.27%)
**Mucinous**	34 (58.62%)	24 (66.67%)	10 (45.45%)
**Endometrioid**	2 (3.45%)	2 (5.56%)	-
**Clear cell**	5 (8.62%)	2 (5.56%)	3 (13.64%)
**Others**	2 (3.45%)	1 (2.78%)	1 (4.55%)
**Mixed**	3 (5.17%)	1 (2.78%)	2 (9.09%)
**Non-epithelial ovarian**			
**Sex cord stromal**	4 (6.06%)	3 (7.32%)	1 (4%)
**Germ cell**	2 (3.03%)	2 (4.88%)	-
**Other**	2 (3.03%)	-	2 (8%)

**Table 3:** Performance of individual classifiers in the identification of ovarian cancer.

Criteria	MIA2G	IOTA-LR2	CA-125	MIA2G and IOTA-LR2	CA-125 and IOTA-LR2
**Sensitivity**					
**%**	91%	80.5%	76.7%	77.4%	69.9%
***n*** **/N**	121/133	107/133	102/133	103/133	93/133
**95% CI**	84.8–95.3%	72.7–86.8%	68.6–83.6%	69.4–84.2%	61.4–77.6%
**Specificity**					
**%**	41.2%	81%	66.7%	85%	88.2%
***n*** **/N**	63/153	124/153	102/153.	130/153	135/153
**95% CI**	33.3.–49.4%	73.9–86.9%	58.6–74.1%	78.3–90.2%	82–92.9%
**Positive Predictive Value**					
**%**	57.3%	78.7%	66.7%	81.7%	83.8%
***n*** **/N**	121/211	107/136	102/153.	103/126	93/111
**95% CI**	50.4–64.1%	70.8–85.2%	58.6–74.1%	73.9–88.1%	75.6–90.1%
**Negative Predictive Value**					
**%**	84%	82.7%	76.7%	81.3%	77.1%
***n*** **/N**	63/75	124/150	102/133	130/160	135/175
**95% CI**	73.7%	75.6–88.4%	68.6–83.6%	74.3–87%	70.2–83.1%
**AUROC**	0.66	0.81	0.72	0.81	0.79
**95% CI**	0.61–0.71	0.76–0.85	0.66–0.77	0.77–0.86	0.74–0.84

**Abbreviations:** CA-125: Cancer antigen-125; IOTA-LR2: The International Ovarian Tumour Analysis—Logistic Regression 2; MIA2G: multivariate index assay.

**Table 4:** Comparative performance for all evaluable women in all cancer cases across menopausal status.

Criteria	MIA2G		IOTA-LR2		IOTA-LR2 and MIA2G		CA-125		IOTA-LR2 and CA-125	
	Pre	Post	Pre	Post	Pre	Post	Pre	Post	Pre	Post
**Sensitivity**										
**%**	86.3%	96.7%	74%	88.3%	69.9%	86.7%	68.5%	86.7%	61.6%	80%
***n*** **/N**	63/73	58/60	54/73	53/60	51/73	52/60	50/73	52/60	45/73	48/60
**95% CI**	76.2%–93.2%	88.5%–99.6%	62.4%–83.5%	77.4%–95.2%	58%–80.1%	75.4%–94.1%	56.6%–78.9%	75.4%–94.1%	49.5%–72.8%	67.7%–89.2%
**Specificity**										
**%**	47.4%	23.1%	86.8%	64.1%	92.1%	64.1%	67.5%	64.1%	94.7%	69.2%
***n*** **/N**	54/114	9/39	99/114	25/39	105/114	25/39	77/114	25/39	108/114	27/39
**95% CI**	37.9%–56.9%	11.1%–39.3%	79.2%–92.4%	47.2%–78.8%	85.5%–96.3%	47.2%–78.8%	58.1%–76%	47.2%–78.8%	88.9%–98%	52.4%–83%
**Positive Predictive Value**										
**%**	51.2%	65.9%	78.3%	79.1%	85%	78.8%	57.5%	78.8%	88.2%	80%
***n*** **/N**	63/123	58/88	54/69	53/67	51/60	52/66	50/87	52/66	45/51	48/60
**95% CI**	42%–60.3%	55%–75.7%	66.7%–87.3%	67.4%–88.1%	73.4%–92.9%	67%–87.9%	46.4%–68%	67%–87.9%	76.1%–95.6%	67.%7–89.2%
**Negative Predictive Value**										
**%**	84.4%	81.8%	83.9%	78.1%	82.7%	75.8%	77%	75.8%	79.4%	69.2%
***n*** **/N**	54/64	9/11	99/118	25/32	105/127	25/33	77/100	25/33	108/136	27/39
**95% CI**	73.1%–92.2%	48.2%–97.7%	76%–90%	60%–90.7%	75%–88.8%	57.7%–88.9%	67.5%–84.8%	57.7%–88.9%	71.6%–85.9%	52.4%–83%
**AUROC**	0.67	0.60	0.80	0.76	0.81	0.75	0.68	0.75	0.78	0.75
**95% CI**	0.61–0.73	0.53–0.67	0..74–0.86	0..68–0.85	0.75–0.87	0.67–0.84	0.61–0.75	0.67–0.84	0.72–0.84	0.66–0.84

**Abbreviations:** CA-125: Cancer antigen-125; IOTA-LR2: The International Ovarian Tumour Analysis—Logistic Regression 2; MIA2G: multivariate index assay; Pre: pre-menopausal; Post: post-menopausal.

**Table 5:** Comparative sensitivity by cancer stage and type.

Findings	Epithelial	Non-Epithelial	Borderline	Early-Stage	Late-Stage
IOTA-LR2	80.2%	83.3%	51.6%	84.8%	97%
***n*** **/N**	97/121	10/12	16/31	56/66	32/33
**95% CI**	71.9%–86.9%	51.6%–97.9%	33.1%–69.8%	73.9%–92.5%	84.2%–99.9%
CA-125	78.5%	58.3%	58.1%	74.2%	97%
***n*** **/N**	95/121	7/12	18/31	49/66	32/33
**95% CI**	70.1%–85.5%	27.7%–84.8%	39.1%–75.5%	62%–84.2%	84.2%–99.9%
MIA2G	92.6%	75%	80.6%	92.4%	97%
***n*** **/N**	112/121	9/12	25/31	61/66	32/33
**95% CI**	86.3%–96.5%	42.8%–94.5%	62.5%–92.5%	83.2%–97.5%	84.2%–99.9%
IOTA-LR2 + CA-125	71.9%	50%	41.9%	70%	93.9%
***n*** **/N**	87/121	6/12	13/31	46/66	31/33
**95% CI**	63%–79.7%	21.1%–78.9%	24.5%–60.9%	57.1%–80.4%	79.8%–99.3%
IOTA-LR2 + MIA2G	78.5%	66.7%	51.6%	80.3%	93.9%
***n*** **/N**	95/121	8/12	16/31	53/66	31/33
**95% CI**	70.1%–85.5%	34.9%–90%	33.1%–69.8%	68.7%–89.1%	79.8%–99.3%

## Data Availability

The data that supports the findings of this study are available in this article.
